# *Vaccinium macrocarpon* Aiton Extract Ameliorates Inflammation and Hyperalgesia through Oxidative Stress Inhibition in Experimental Acute Pancreatitis

**DOI:** 10.1155/2018/9646937

**Published:** 2018-05-14

**Authors:** Danielle Gomes Santana, Alan Santos Oliveira, Marília Trindade de Santana Souza, José Thiago do Carmo Santos, Neuza Mariko Aymoto Hassimotto, Ana Mara de Oliveira e Silva, Renata Grespan, Enilton Aparecido Camargo

**Affiliations:** ^1^Department of Physiology, Federal University of Sergipe, 49100-000 São Cristóvão, SE, Brazil; ^2^Department of Food Science and Experimental Nutrition, School of Pharmaceutical Sciences, University of São Paulo, 05508-000 São Paulo, SP, Brazil; ^3^Food Research Center (FoRC), Research Innovation and Dissemination Centers-São Paulo Research Foundation (CEPID-FAPESP), 05508-080 São Paulo, SP, Brazil; ^4^Department of Nutrition, Federal University of Sergipe, 49100-000 São Cristóvão, SE, Brazil

## Abstract

We evaluated the effect of the hydroethanolic extract of fruits of* Vaccinium macrocarpon* (HEVm) in a model of acute pancreatitis (AP) in mice. AP was induced by two injections of L-arginine and animals were treated with HEVm (50, 100, and 200 mg/kg, p.o.) or vehicle (saline) every 24 h, starting 1 h after the induction of AP. Phytochemical analysis of the extract and measurement of inflammatory and oxidative stress parameters, as well as abdominal hyperalgesia, were performed. Catechin, epicatechin, rutin, and anthocyanins were identified in HEVm. Treatment with HEVm decreased L-arginine-induced abdominal hyperalgesia (from 48 to 72 h). Also, treatment with HEVm decreased L-arginine-induced pancreatic edema, pancreatic and pulmonary neutrophil infiltration, and levels of tumor necrosis factor-*α*, interleukin-1*β*, and interleukin-6, after 72 h of induction. L-arginine-induced hyperamylasemia and hyperlipasemia were also reduced by the treatment with HEVm in comparison to vehicle-treated group. Moreover, lipoperoxidation, carbonyl radicals, nonprotein sulfhydryl groups, and activity of catalase and superoxide dismutase, but not glutathione peroxidase, were restored by the treatment with HEVm. These results show that treatment with HEVm decreased hyperalgesia and pancreatic/extrapancreatic inflammation and oxidative damage in L-arginine-induced AP, making this extract attractive for future approaches designed to treat this condition.

## 1. Introduction

Acute pancreatitis (AP) is a disease that leads to pancreatic inflammation, related to the premature activation of digestive enzymes. The incidence of this disease has increased in recent years [1] which has contributed to a significant increase in mortality, mainly caused by complications of this condition such as the systemic inflammatory response [2].

The treatment of AP is primarily symptomatic and based on the initial evaluation of disease severity. It is directed to control pain, promote supportive therapy, meet the nutritional needs, treat complications, and prevent pancreatic infection and spread of the inflammatory process to remote organs [3]. The lack of specific therapy for AP along with the high mortality rates observed in severe cases makes experimental studies to find new treatment alternatives of great interest.

Among the sources of new medicinal active principles, natural products with proven antioxidant potential are of interest for the treatment of AP, since the local release of reactive oxygen species (ROS) and inflammatory cell infiltration in the pancreas play a key role in the development of AP and complications associated with this condition [4].

The fruit of* Vaccinium macrocarpon*, Aiton (Ericaceae), is popularly known as cranberry and is used to make juices and sauces throughout North America. Due to promising results, the medicinal use of this plant has spread throughout to the Western world [5]. It contains anthocyanins, flavonoids, proanthocyanidins, condensed tannins, and phenolic acids. Recent studies have shown that an extract of fruits of* V. macrocarpon* containing approximately 1.82% anthocyanins can attenuate hepatic inflammation in obese mice [6] and inhibit the adhesion of pathogenic* E. coli* in patients with recurrent urinary tract infection [7]. In addition, treatment with this extract led to decreased intestinal inflammation in obese mice [5]. Clinically, cranberry preparations have been used in the treatment of urinary tract diseases and vascular inflammation, demonstrating high therapeutic potential [8], which may still be underestimated.

The need for new therapeutic options to treat AP along with the potential anti-inflammatory and antioxidant effect presented by cranberry makes it a viable alternative for the treatment pancreatitis. Thus, we investigated the possible effect of a hydroethanolic extract of fruits of* Vaccinium macrocarpon* (HEVm) in experimental AP in mice.

## 2. Material and Methods

### 2.1. Plant Material

The hydroethanolic extract of fruits of* Vaccinium macrocarpon* (HEVm) was purchased from Fagron BV, lot number 14083757D, with certificate of quality control analysis (HHHC140402).

### 2.2. Characterization of Hydroethanolic Extract of Fruits of* Vaccinium macrocarpon*

Samples were dissolved with methanol : water (70 : 30, v : v), acidified with 5% acetic acid, using an ultrasound bath (Ultrasonic Cleaner 8892E-DTH; Cole-Parmer, Illinois, USA). After 10 min at room temperature, the dissolved extract was centrifuged at 3,500 ×g, for 10 min at 4°C. This procedure was repeated 3 times and the supernatants were combined. The supernatants were reduced under vacuum at 40°C in a rotary evaporator (Rotavapor RE 120; Buchi, Flawil, Switzerland) prior to passage through a C-18 extraction column (1 g) (Supelclean LC-18, Supelco, Pennsylvania, USA) previously conditioned with methanol and ultrapure water. The sample was loaded into the column and washed with ultrapure water. The phenolic compounds were eluted with methanol acidified with 0.3% HCl. The eluates were completely dried using a rotary evaporator under vacuum at 40°C, resuspended in methanol acidified with 5% acetic acid, and filtered through a 0.45 *μ*m PVDF filter (Millipore Ltd.) for the quantification of flavonoids by high-performance liquid chromatography-diode array detection (HPLC-DAD) analysis.

Quantification of flavonoids was carried out using an Agilent 1260 Infinity Quaternary LC System (Agilent Technologies, USA) coupled to a diode array detector (DAD). The column used was a 5 *μ*m Prodigy ODS3 column (4.60 × 250 mm) (Phenomenex Ltd., UK) with a flow rate of 1 mL/min at 25°C. The mobile phase consisted of water/formic acid/acetonitrile (96 : 1 : 3 solvent A, and 58 : 1 : 51 solvent B). The solvent gradient was 10% B at the beginning, 25% at 10 minutes, 31% at 15 minutes, 40% at 20 minutes, 50% at 30 minutes, 100% at 40 minutes, 10% at 45 minutes, and 10% at 50 minutes. Chromatograms were acquired at 270, 370, and 525 nm. The flavonoids were identified by comparing their retention times, diode array spectral characteristics, and mass spectra, analyzed using LC-ESI-MS/MS, with the standards and the data available in the literature. The quantification was done using a calibration curve of the commercial standards (catechin, epicatechin from Sigma-Aldrich (St. Louis, MO, USA), and cyanidin-3-O-glucoside from Extrasynthese, Genay, France).

The identification of the flavonoids was performed using a Prominence Liquid Chromatograph (Shimadzu, Japan) linked to an AmaZon SL ion trap mass spectrometer (Bruker Daltonics, Bremen, Germany) with an electrospray ionization (ESI) interface. The solvent gradient conditions were the same as those used for the HPLC-DAD analysis. After the sample was passed through the DAD, the flow rate was changed to 0.8 mL/min for examination by the mass spectrometer. The ESI was used in the positive and negative mode. The mass spectrometer operating condition was collision energy of 4,500 V for positive and negative mode. The analysis was carried out using a full scan from *m*/*z* 100 to 1,500. The compounds were identified according to mass spectral characteristics and comparison with literature data.

### 2.3. Animals

Male adult Swiss mice (30–35 g) were obtained from the Animal Center of Federal University of Sergipe. The animals were maintained at 21 ± 2°C with free access to food (Nuvilab®) and filtered water under a 12 : 12 h light/dark cycle. All experimental procedures were approved by the Ethics Committee for Animal Use in Research of Federal University of Sergipe (protocol number 09/16) and were conducted in compliance with the Guide for Care and Use of Laboratory Animals (National Institutes of Health). During the experiments, animals were randomly distributed among the experimental groups and the experimenter was unaware of each group's identification.

### 2.4. Experimental Procedure

Pancreatitis induction was performed by two intraperitoneal (i.p.) injections, 1 h apart, of L- arginine at the dose of 4 g/kg [9]. The solution for injection contained 8% L-arginine in sterile saline with pH adjusted to 7.0. The control group received sterile saline (NaCl, 0.9%). Seventy-two hours after the last injection, the animals were anesthetized (80 mg/kg of ketamine plus 10 mg/kg of xylazine), euthanized by exsanguination, and transcardially perfused with saline plus heparin (5 IU/L), and tissue samples (pancreas and lung) were collected. Blood was centrifuged at 1,000 ×g for 15 min at room temperature to separate serum samples. Aliquots of serum and tissues were stored at −80°C for proper analysis.

### 2.5. Experimental Design

Animals were randomly distributed into five groups (*n* = 5-6 each). In the saline + vehicle group, animals were injected with sterile saline (0.9%, 5 mL/kg; i.p.; administration for 2 times) and treated with the vehicle (sterile saline; i.p.) at 1, 25, and 49 hours after the last injection of saline. In the L-arginine + vehicle group, animals were injected with L-arginine (4 g/kg; i.p.; administration for 2 times) and treated with the vehicle (i.p.) at 1, 25, and 49 hours after the last injection of L-arginine. In the three AP + HEVm groups, animals were injected with L-arginine (4 g/kg; i.p.; administration for 2 times) and treated with HEVm (50, 100, or 200 mg/kg p.o.) at 1, 25, and 49 hours after the last injection of L-arginine.

Additional control groups were treated with dexamethasone (5 mg/kg, s.c.) at 1, 25, and 49 hours after the last injection of L-arginine (control for inflammatory parameters) or morphine (5 mg/kg, i.p., 30 min before each measurement performed at 24, 48, and 72 h; control for abdominal hyperalgesia) [10].

### 2.6. Measurement of Abdominal Hyperalgesia

To evaluate the mechanical hyperalgesia in the abdominal region of mice, we used an electronic von Frey apparatus (Insight, Ribeirão Preto, São Paulo, Brazil), according to Abreu et al. [11], with minor adaptations. On the day of testing, mechanical stimuli were applied to the anterior lateral region of the abdomen of the animals, in triplicate, prior to any manipulation and 24, 48, or 72 h after AP induction. At each time point, an increasing stimulus (in g) was applied to the abdominal region of the mice, with an interval of at least 1 min apart, until any withdrawal behavior was observed and the threshold force (0.1 to 1000 g) value was registered by the equipment.

This test was performed off-stage, so that the examiner did not know the identity of the groups. Data were expressed as variation (Δ) by subtracting the mean value obtained from the three measurements taken at the time point referred to after AP induction from the mean basal value recorded for each animal (prior to pancreatitis).

### 2.7. Evaluation of Locomotor Activity

The locomotor activity of mice was investigated in a circular open field apparatus (60 cm diameter; Insight, Ribeirão Preto, São Paulo, Brazil) as previously described [12]. The animals (*n* = 5) were pretreated with HEVm (200 mg/kg) or vehicle (1 h before) or diazepan (1 mg/kg, 30 min before) and were individually placed in the apparatus. After 1 min, the distance traveled (m), mobility time (s), and number of lines crossed were registered over a period of 5 min.

### 2.8. Determination of Inflammatory Markers

The edema index was calculated as the ratio of wet weight and dry weight of the samples of pancreatic tissue as previously described [10].

To determine myeloperoxidase (MPO) activity, pancreas and lung samples were collected and homogenized with potassium phosphate buffer (50 mM, pH 6.0 containing 0.5% hexadecyltrimethylammonium bromide). Aliquots of the homogenates were centrifuged (2 min, 8,000 ×g, 4°C) and aliquots of the supernatants were incubated with a solution of* o*-dianisidine hydrochloride (0.167 mg/mL containing 0.005% H_2_O_2_). The MPO activity was measured kinetically in a microliter plate scanner (Biotek®) at 460 nm over a period of 5 min. Results were expressed as units of MPO per mg tissue (UMPO/mg tissue). UMPO was considered as the amount of enzyme that degrades 1 mmol of hydrogen peroxide/min [13].

Enzyme-linked immunosorbent assay (ELISA) for interleukin- (IL-) 6, IL-1*β*, and tumor necrosis factor- (TNF-) *α* was carried out in homogenate samples of pancreas and lung according to the manufacturer's instructions (R&D Systems). The results were corrected by the amount of tissue protein and expressed as pg of each cytokine/mg of protein. The protein content of tissues was determined by the Bradford method using the Bio-Rad® protein assay reagent.

### 2.9. Determination of Biochemical Parameters in Serum

Biochemical parameters in serum were assessed for each experimental group by using specific commercial kits for amylase (Katal Biotechnology, Belo Horizonte, MG, Brazil), lipase (Human do Brasil, São Paulo, SP, Brazil), aspartate aminotransferase (AST), and alanine aminotransferase (ALT) (Liquiform Labtest, Lagoa Santa, MG, Brazil), according to each manufacturer's instructions.

### 2.10. Determination of Antioxidant Activity

Samples of pancreas and lung were homogenized in phosphate buffer 50 mM pH 7.0, centrifuged at 10,000 ×g, and the supernatant was used for all antioxidant assays.

Lipid peroxidation was determined by the presence of thiobarbituric acid reactive substances in samples of pancreas and lung homogenates by the method previously described by Bose et al. [14], with minor modifications [10]. The results were expressed as pmol of malondialdehyde (MDA) formed per mg of protein.

Pancreatic and lung nonprotein sulfhydryl groups (NP-SH) were determined by Ellman's reaction using 5′5′-dithio-bis-2-nitrobenzoic acid, as previously described [15]. Results were expressed as *μ*g of NP-SH/mg of protein.

The determination of oxidized protein content (carbonyl groups) was performed [16] by the reaction of carbonyl groups and 2,4-dinitrophenylhydrazine. The concentration of carbonyl groups was calculated by using 21.5 mM^-1 ^cm^−1^ as the extinction coefficient for aliphatic hydrazones and the results were expressed as nmol of carbonyls/mg of protein.

Tissue antioxidant status was evaluated in the homogenate of pancreas and lung tissues using the ferric reducing/antioxidant power (FRAP) assay [17]. Results were plotted against a standard curve of ferrous sulphate (500–1500 *μ*mol/L) and were expressed as *μ*mol of ferrous sulphate/mg of protein.

Catalase (CAT) activity was appraised by the decrease in absorption of H_2_O_2_ at 240 nm, due to H_2_O_2_ consumption by CAT, as previously described [18]. Specific activity was expressed as units of CAT/mg of protein.

The activity of Cu/Zn SOD was assayed by the adrenaline method, based on the capacity of SOD to inhibit autoxidation of adrenaline to adrenochrome. One unit of SOD activity was defined as the amount of protein causing 50% inhibition of the autoxidation of adrenaline at 26°C [19].

Glutathione peroxidase (GSH-Px) activity was determined as described previously [20]. Enzyme activity was expressed as *μ*mol of glutathione oxidized/mg of protein/min by using the extinction coefficient for NADPH (6220 M^-1 ^cm^−1^).

### 2.11. Statistical Analysis

Results were expressed as mean ± standard error of mean (SEM) and analyzed by one- or two-way analysis of variance (ANOVA) followed by Bonferroni's post hoc test, using the GraphPad Prism software (version 5.0). *p* < 0.05 was considered significant.

## 3. Results

### 3.1. Phytochemical Analysis of* HEVm*

**Figure 1:**
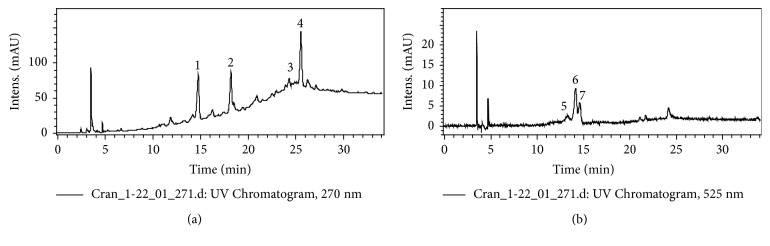
Chromatograms obtained by HPLC-DAD at *λ* = 270 nm (a) and 525 nm (b) for the hydroethanolic extract of fruits of* Vaccinium macrocarpon* (HEVm). The identification of the peaks is given in Table 1.

Figure 1 shows the HPLC-DAD chromatograms of the HEVm extract and the respective identification of the compounds by LC-ESI-MS/MS is shown in Table 1. Seven compounds were identified (three flavan-3-ols, one flavonol, and three anthocyanins). The major compounds were catechin and epicatechin (Figure 1(a), peaks 1 and 2, *m*/*z* 291). The main anthocyanins were cyanidin-3-O-glucoside (*m*/*z* 449, peak 6, Figure 1(b)) followed by cyanidin-coumaroyl-hexoside (*m*/*z* 595, peak 7, Figure 1(b)), both with characteristic molecular ions of cyanidin (*m*/*z* 287). The total flavonoids content in powdered cranberry was 875.65 mg/100 g, where flavan-3-ol contributed 93.3%, followed by flavonol (4.3%) and anthocyanins (2.4%).

### 3.2. Effect of* HEVm* on Inflammatory Parameters

**Figure 2:**
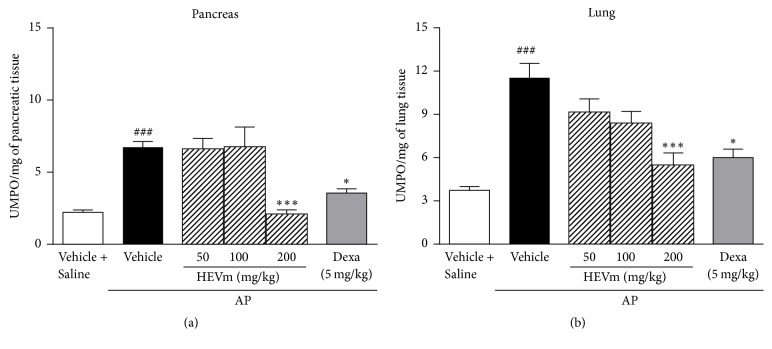
Effect of the hydroethanolic extract of fruits of* Vaccinium macrocarpon* (HEVm) on pancreatic and lung myeloperoxidase (MPO) activity. Mice were submitted to injection of L-arginine or saline, treated with HEVm (50, 100, or 200 mg/kg), dexamethasone (Dexa; 5 mg/kg), or vehicle (saline), and euthanized after pancreatitis induction. MPO activity was measured in pancreas (a) and lung (b) tissues (*n* = 5-6). ^###^*p* < 0.001 versus vehicle + saline and ^*∗*^*p* < 0.05 or ^*∗∗∗*^*p* < 0.001 versus vehicle + L-arginine. One-way ANOVA followed by Bonferroni's test.

Pancreatic inflammation induced by L-arginine was characterized by a marked increase of pancreatic MPO activity after 72 h. The treatment with HEVm at 200 mg/kg prevented this effect (*p* < 0.001) (Figure 2(a)). In addition, the induction of pancreatitis significantly increased MPO activity in the lung tissue compared with the saline group. This effect was also reduced by the treatment with HEVm at 200 mg/kg (*p* < 0.001, Figure 2(b)). L-arginine administration enhanced pancreatic edema index after injection (5.15 ± 0.05, *p* < 0.001) compared with the saline group (4.39 ± 0.08), and the treatment with HEVm at 200 mg/kg produced a significant inhibition of this effect (4.38 ± 0.08, *p* < 0.001), as did dexamethasone (4.38 ± 0.09, *p* < 0.001). However, the treatment with HEVm at 50 or 100 mg/kg did not alter edema (5.14 ± 0.14, 4.92 ± 0.14, resp.).

**Figure 3:**
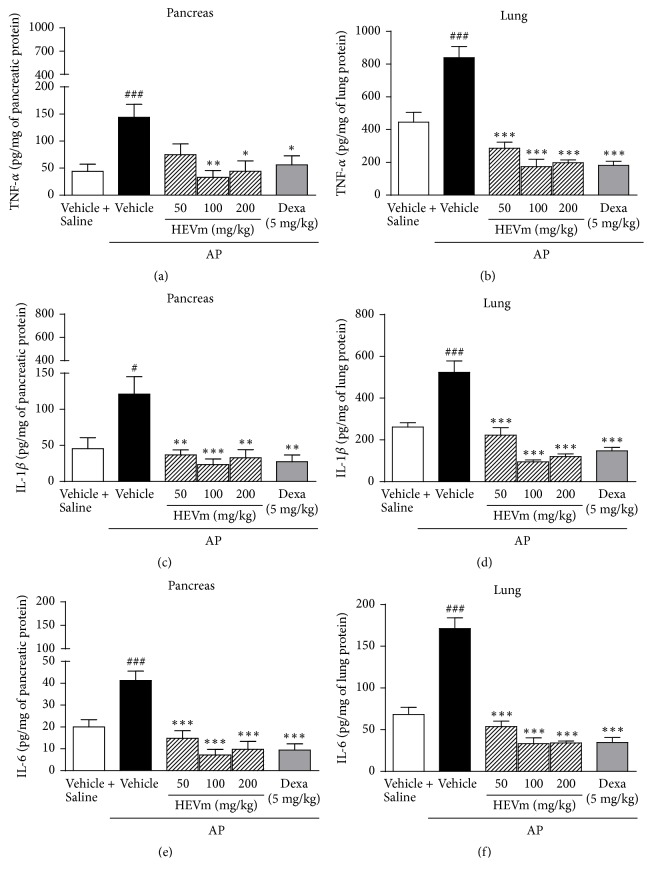
The hydroethanolic extract of fruits of* Vaccinium macrocarpon* (HEVm) inhibits tumor necrosis factor- (TNF-) *α*, interleukin- (IL-) 1*β*, and IL-6 levels in pancreas and lung. Mice were submitted to injection of L-arginine or saline, treated with HEVm (50, 100, or 200 mg/kg), dexamethasone (Dexa; 5 mg/kg), or vehicle (saline), and euthanized after pancreatitis induction. TNF-*α*, IL-1*β*, and IL-6 levels were measured in pancreas ((a), (c), and (e), resp.) and lung ((b), (d), and (f) resp.) tissues (*n* = 5-6). ^#^*p* < 0.05 or ^###^*p* < 0.001 versus vehicle + saline and ^*∗*^*p* < 0.05, ^*∗∗*^*p* < 0.01, or ^*∗∗∗*^*p* < 0.001 versus L-arginine + vehicle. One-way ANOVA followed by Bonferroni's test.

In addition to the increase of the above-mentioned inflammatory parameters, the levels of TNF-*α*, IL-1*β*, and IL-6 were significantly increased in pancreas and lung after AP induction by L-arginine (Figures 3(a)–3(f)) compared with animals injected with saline. The treatment with HEVm strongly modulated the levels of these cytokines. Levels of TNF-*α* were significantly decreased by the treatment with HEVm in the pancreatic (*p* < 0.01 and *p* < 0.05, resp., at 100 and 200 mg/kg, Figure 3(a)) and lung tissue (*p* < 0.001 at 50, 100, and 200 mg/kg, Figure 3(b)) compared with vehicle-treated animals. The amount of IL-1*β* was also lower in pancreas (*p* < 0.01 at 50 and 200 mg/kg and *p* < 0.001 at 100 mg/kg, Figure 3(c)) and lung tissues (*p* < 0.001, Figure 3(d)) in the group treated with HEVm compared to the vehicle-treated animals. A similar effect was observed for IL-6 levels, for which the groups treated with HEVm presented significantly lower levels of this cytokine in pancreas (*p* < 0.001, Figure 3(e)) and lung (*p* < 0.001, Figure 3(f)) compared with vehicle-treated animals. As a control, treatment with dexamethasone decreased the levels of these cytokines in both pancreas and lungs tissues (Figure 3).

### 3.3. Effect of* HEVm* on Mechanical Abdominal Hyperalgesia

**Figure 4:**
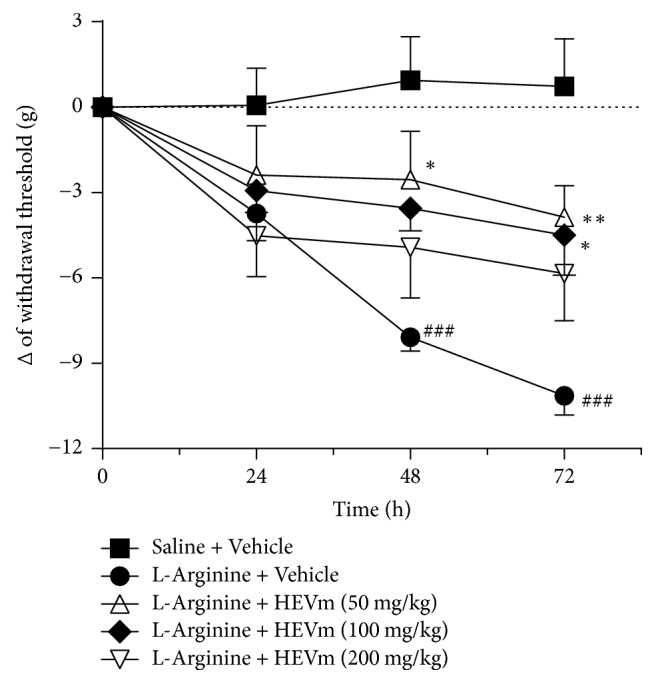
The hydroethanolic extract of fruits of* Vaccinium macrocarpon* (HEVm) reduces abdominal hyperalgesia in L-arginine-induced pancreatitis. Animals were submitted to injection of L-arginine or saline, treated with HEVm (50, 100, or 200 mg/kg) or vehicle (saline), and euthanized after pancreatitis induction. The variation (Δ) of the threshold force to cause withdrawal behavior was calculated by subtracting each time point from basal values (0 h) and was expressed in g for the intensity of stimulus for *n* = 5. ^###^*p* < 0.001 versus the respective saline + vehicle group; ^*∗*^*p* < 0.05 or ^*∗∗*^*p* < 0.01 versus the respective L-arginine + vehicle. Two-way ANOVA (interaction of treatment versus time: *p* < 0.001 and* F*_(15,81)_ = 3,712) followed by Bonferroni's test.

L-arginine-induced AP was accompanied by a marked reduction of the intensity of stimulus needed to cause withdrawal behavior in mice after 48 or 72 h, denoting that mice developed abdominal hyperalgesia as a consequence of the pancreatic inflammation. Two-way ANOVA indicated a significant interaction between the time and treatment in this experiment (*p* < 0.001;* F* = 3.712). The injection of saline instead of L-arginine did not alter the mice's withdrawal behavior (Figure 4) in comparison to basal evaluation.

Figure 4 also demonstrates that the treatment with HEVm clearly reduced the abdominal hyperalgesia of mice submitted to AP after 48 h (*p* < 0.01 at 50 mg/kg and *p* < 0.05 at 100 mg/kg) or 72 h (*p* < 0.001 at 50 mg/kg, *p* < 0.01 at 100 mg/kg, and *p* < 0.05 at 200 mg/kg).

As an experimental control, treatment with morphine inhibited abdominal hyperalgesia stimulus at 24 (9.7 ± 1 g; *p* < 0.001), 48 (8.3 ± 2 g; *p* < 0.001), and 72 h (5.8 ± 2 g; *p* < 0.001) after induction of pancreatitis compared with animals treated with the vehicle (−3.7 ± 1, −8.1 ± 1, and −10.1 ± 1 g, resp., at 24, 48, and 72 h after AP).

Of interest, no difference was observed in the mean basal values of intensity of stimulus needed to induce withdrawal behavior among the groups (saline + vehicle: 25.9 ± 2.0 g; L-arginine + vehicle: 28.2 ± 0.7 g; L-arginine + HEVm [50 mg/kg]: 26.5 ± 1.0 g; L-arginine + HEVm [100 mg/kg]: 26.1 ± 0.3 g; L-arginine + HEVm [200 mg/kg]: 28.0 ± 0.8 g; and L-arginine + morphine: 27.1 ± 0.6 g).

### 3.4. Lack of Effect of* HEVm* on the Locomotor Activity

Pretreatment of mice (*n* = 5) with HEVm (200 mg/kg) did not modify the distance traveled (34.3 ± 3.1 m), mobility time (258 ± 17 s), or the number of lines crossed (238 ± 17) when compared to the vehicle group (36.3 ± 4.1 m, 275 ± 9 s, and 245 ± 20, resp.). As a control, pretreatment with diazepan decreased these parameters significantly (8.4 ± 3.6 m, 17 ± 5 s, and 13 ± 3; *p* < 0.001 each) in comparison to the vehicle-treated group.

### 3.5. Effect of HEVm on Serum Biochemical Parameters

L-arginine-induced AP significantly increased the serum levels of amylase and lipase. This effect was accompanied by increase of the serum concentrations of ALT and AST after the induction of AP compared with saline-injected animals. Treatment with HEVm reduced the serum levels of amylase (200 mg/kg, *p* < 0.001), lipase (200 mg/kg, *p* < 0.01), ALT (100 mg/kg, *p* < 0.05; 200 mg/kg, *p* < 0.05), and AST (100 mg/kg, *p* < 0.05; 200 mg/kg, *p* < 0.01). Treatment with dexamethasone (5 mg/kg) decreased the levels of amylase (*p* < 0.01) and lipase (*p* < 0.05) in serum (Table 2).

### 3.6. Effect of* HEVm* on Oxidative Stress Markers and Antioxidant Enzymes

**Figure 5:**
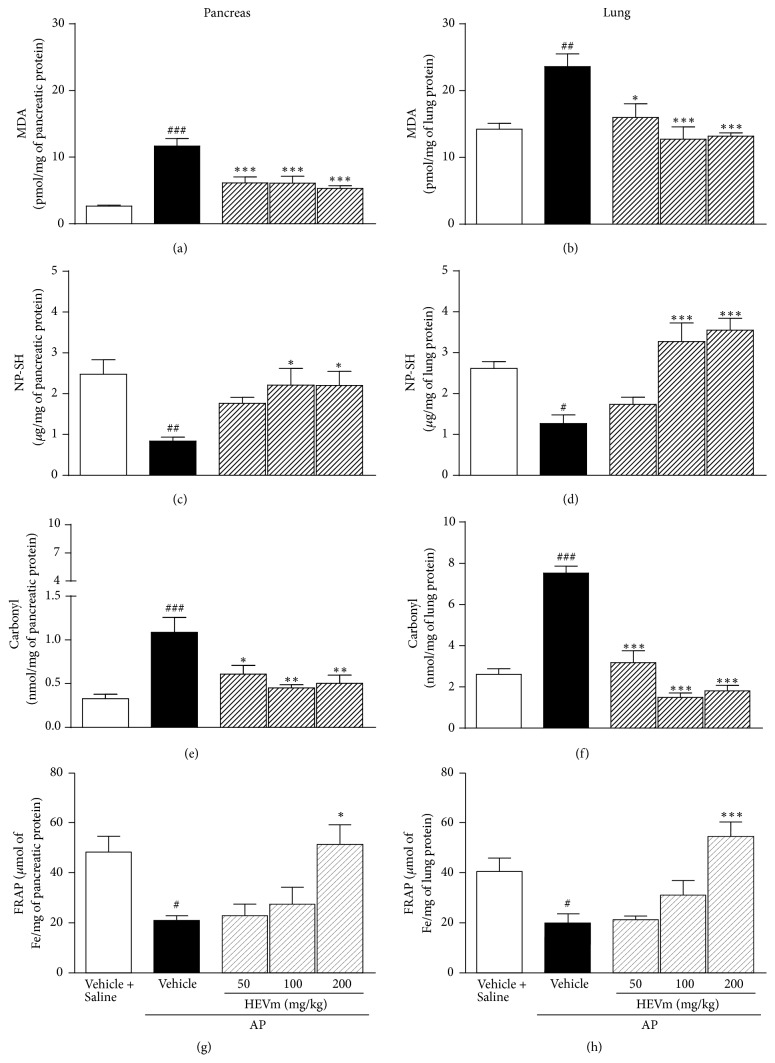
Effect of the hydroethanolic extract of fruits of* Vaccinium macrocarpon* (HEVm) on pancreatic and lung biomarkers of oxidative stress. Animals were submitted to injection of L-arginine or saline, treated with HEVm (50, 100, or 200 mg/kg) or vehicle (saline), and euthanized 72 h after pancreatitis induction. Malondialdehyde (MDA) concentrations, nonprotein sulfhydryl groups (NP-SH), carbonyl groups, and ferric reducing/antioxidant power (FRAP) were measured in pancreas ((a), (c), (e), and (g), resp.) and lung ((b), (d), (f), and (h), resp.) tissues (*n* = 5-6). ^#^*p* < 0.05, ^##^*p* < 0.01, and ^###^*p* < 0.001 versus vehicle + saline; ^*∗*^*p* < 0.05, ^*∗∗*^*p* < 0.01, and ^*∗∗∗*^*p* < 0.001 versus vehicle + AP (one-way ANOVA followed by Bonferroni's test).

Lipid peroxidation products, in particular MDA, were higher in both pancreas (Figure 5(a)) and lung (Figure 5(b)) after the induction of pancreatitis. This effect was inhibited by the treatment with HEVm at doses of 50, 100, and 200 mg/kg (*p* < 0.001; Figure 5(a)) in pancreatic tissue compared with animals treated with the vehicle. In lung tissue, the production of MDA was also reduced by the doses of 50 (*p* < 0.05), 100, and 200 mg/kg (*p* < 0.001; Figure 5(b)) compared with animals treated with the vehicle.

In the pancreas and lung tissue of animals with AP, higher amounts of carbonyl radicals were found (Figures 5(c) and 5(d)). This effect was reversed in pancreas (*p* < 0.05 at 50 mg/kg and *p* < 0.01 at 100 and 200 mg/kg; Figure 5(c)) and lung (*p* < 0.001 for all doses; Figure 5(d)) of animals treated with HEVm.

In addition, pancreatic and lung nonprotein sulfhydryl groups (NP-SH) were decreased by AP induction (Figures 5(e) and 5(f)). Treatment with HEVm at the doses of 100 and 200 mg/kg maintained the NP-SH content in pancreas (*p* < 0.05, Figure 5(e)) and lung (*p* < 0.001, Figure 5(f)) compared with animals treated with the vehicle.

Additionally, tissue antioxidant status was measured in the pancreas and lung through the determination of the total antioxidant activity by the reduction of iron (FRAP). Figures 5(g) and 5(h) demonstrate that tissue antioxidant capacity was significantly reduced in pancreas and lung after AP induction and the treatment with 200 mg/kg of HEVm maintained this capacity near the levels observed in animals from the vehicle + saline group, in both pancreas (*p* < 0.05; Figure 5(g)) and lung (*p* < 0.001; Figure 5(h)).

**Figure 6:**
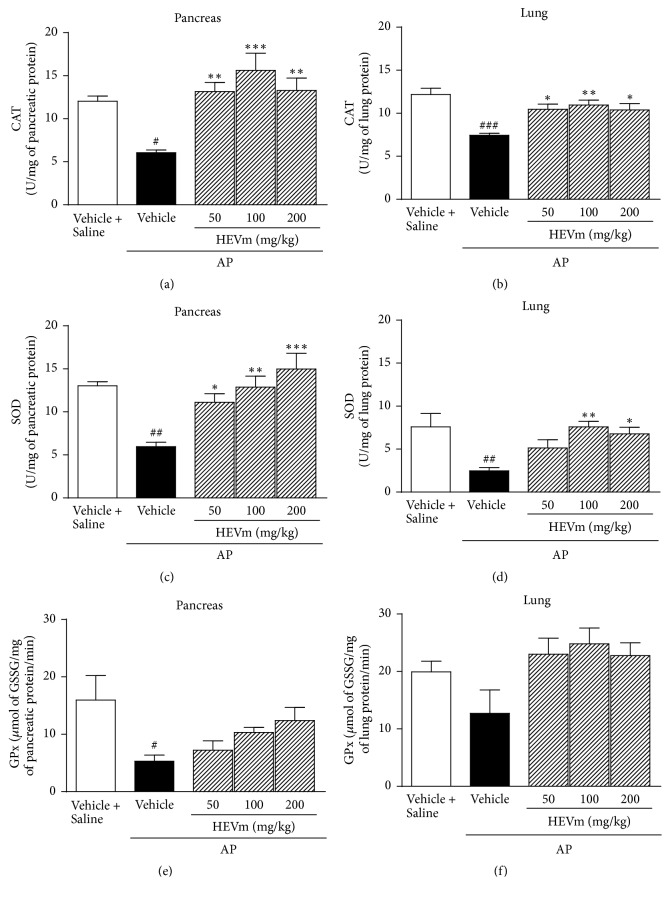
*Effect of the hydroethanolic extract of fruits of Vaccinium macrocarpon (HEVm) on activity of antioxidant enzymes in pancreas and lung*. Animals were submitted to injection of L-arginine or saline, treated with HEVm (50, 100, or 200 mg/kg) or vehicle (saline), and euthanized 72 h after pancreatitis induction. Catalase (CAT), superoxide dismutase (SOD), and glutathione peroxidase (GPx) activity were measured in pancreas ((a), (c), and (e), resp.) and lung ((b), (d), and (f), resp.) tissues (*n* = 5-6). ^#^*p* < 0.05, ^##^*p* < 0.01, and ^###^*p* < 0.001 versus vehicle + saline; ^*∗*^*p* < 0.05, ^*∗∗*^*p* < 0.01, and ^*∗∗∗*^*p* < 0.001 versus vehicle + AP (one-way ANOVA followed by Bonferroni's test).

Since the treatment with HEVm produced a marked antioxidant effect in pancreas and lung tissue of animals with pancreatitis, the contribution of some antioxidant enzymes to this action was studied. The induction of AP reduced the activity of CAT, SOD, and GSH-Px enzymes in pancreas and CAT and SOD activities in lung (Figure 6).  Figure 6(a) shows the increase in pancreatic CAT activity of mice treated with HEVm at doses of 50 mg/kg (*p* < 0.01), 100 mg/kg (*p* < 0.001), and 200 mg/kg (*p* < 0.01). A similar effect was observed in lungs (Figure 6(b)) of animals treated with HEVm at doses of 50 (*p* < 0.05), 100 (*p* < 0.01), and 200 mg/kg (*p* < 0.05). Concomitantly, the activity of SOD significantly increased in the pancreas after treatment with HEVm at doses of 50 (*p* < 0.05), 100 (*p* < 0.01), and 200 mg/kg (*p* < 0.001; Figure 6(c)), which was also observed in lung tissue after treatment with 100 (*p* < 0.01) and 200 mg/kg (*p* < 0.05; Figure 6(d)). However, GPx activity was not modified by the treatment with HEVm in both pancreas and lung tissues (Figures 6(e) and 6(f)).

## 4. Discussion

The need for new experimental alternatives to treat AP is still a challenge, since there are no specific treatments for clinical management of this disease [3]. In this study we present data that demonstrates that HEVm can modulate the inflammatory response in pancreas and lung (with the participation of oxidative stress) and abdominal hyperalgesia during L-arginine-induced AP. The finding that HEVm has beneficial effect in experimental pancreatitis is novel for this extract, which is frequently used by people to treat inflammatory conditions of the urinary system [8]. However, the presence of flavonoids in this extract supports the possibility of a beneficial effect on AP. For this reason, we used the hydroethanolic extract of cranberry. Our current data reinforce this hypothesis in the model of pancreatitis induced by L-arginine.

The phytochemical analysis of HEVm showed that catechins and epicatechins are its major components. It was possible to identify three flavan-3-ols, one flavonol, and three anthocyanins. Catechin, epicatechins, and cyanidin derivatives were previously described in cranberry preparations [21, 22], as was quercetin-3-O-rutinoside (rutin) [23]. These compounds exert various biological effects, including antioxidant and anti-inflammatory effects [11, 21].

Treatment with HEVm induced protective effects against pancreatic inflammation. The activity of MPO, edema index, and production of cytokines (TNF-*α*, IL-1*β*, and IL-6) were reduced by this extract. Along with the lower pancreatic damage in animals treated with HEVm, the levels of serum amylase and lipase were lower, which corroborates the reduction of pancreatic damage. Neutrophil infiltration is a hallmark of AP [24] and our data show that HEVm can interfere with the presence of these cells in the pancreas. This agrees with previous findings that cranberry extract reduces the MPO activity in mice paws injected with carrageenan [23]. Similarly, treatment with 1.5% dried whole cranberry powder (incorporated in the diet) reduced MPO activity in the colon of mice submitted to dextran sulphate sodium-induced colitis [25]. Altogether, these results highlight the anti-inflammatory effect of the extract of fruits of* V. macrocarpon*. In accordance with this effect, treatment with HEVm produced antiedematogenic effect. Corroborating this finding, [23] showed that the extract of* V. macrocarpon* fruits reduces the production of prostaglandins* in vitro*, possesses antioxidant activity, and inhibits carrageenan-induced mice paw edema. The reduction of neutrophil migration to the pancreas and edema formation induced by HEVm certainly are dependent on the inhibitory activity of proinflammatory cytokines. Our results demonstrate that pancreatic contents of TNF-*α*, IL-1*β*, and IL-6 were reduced by the treatment with HEVm. Other studies have also demonstrated that the extract of cranberry inhibited IL-6, IL-8, and prostaglandin E_2_ production by gingival fibroblasts stimulated with lipopolysaccharide* in vitro* [26] and that serum IL-1*β* and interferon-*γ* concentrations and TNF-*α* and IL-1*β* mRNA expression in colon were reduced by treatment with cranberry in mice submitted to dextran sulphate sodium-induced colitis [25].

Besides pancreatic inflammation, abdominal hyperalgesia was observed 48 and 72 hours after pancreatitis induction. Similar results were described by Abreu et al. [11] in the model of L-arginine-induced pancreatitis and we believe that this mimics the abdominal pain observed in humans after an AP attack. The reduction of AP-induced abdominal hyperalgesia by the treatment with HEVm may be a consequence of the decrease of pancreatic inflammatory mediators, such as TNF-*α*, IL-1*β*, and IL-6. However, we cannot discard the possibility that substances present in HEVm blocked the stimulation of nociceptive pathways. Since HEVm administration did not alter the locomotor activity of mice, we can exclude the possibility of muscular relaxation or central nervous system depression that could bias the nociceptive measurement. Few studies have focused on the possible analgesic effect of* V. macrocarpon *fruits. A randomized clinical study demonstrated that men receiving radiation therapy for prostate cancer and taking cranberry capsules presented lower incidence of pain and burning [27]. The presence of flavonoids could also contribute to this antihyperalgesic effect. A previous study showed that the extract of grapefruit rich in proanthocyanidin (a class of flavonoids) reduced nociception caused by sciatic nerve ligation-induced neuropathic pain in rats [28]. In addition, the grapefruit extract reduced the nociception induced by formalin in mice, by mechanisms involving the participation of opioid receptor activation [29]. Concordantly, a previous study by our group showed that rutin reduced abdominal hyperalgesia in L-arginine-induced pancreatitis in mice [11].

Following AP induction, many extrapancreatic responses were observed in animals injected with L-arginine, such as the increased MPO activity in lung and increased markers of hepatic injury. Although the mechanisms underlying these alterations are not well understood in this model of pancreatitis, the beneficial effect of HEVm is supported by these results. Serum ALT and AST levels were used as markers of extrapancreatic alterations in the present study. The increase in ALT, but not AST, is usually predictive of biliary pancreatitis [30]. However, in the model of L-arginine-induced AP in rats, other authors have shown that both AST and ALT are increased [31], but no study has previously measured these enzymes in mice. Interestingly, this increase in hepatic enzymes was partially reduced by the administration of HEVm, which may in part be due to the reduction of pancreatic lesions, as observed by the decrease of pancreatic inflammatory markers and enzymes (amylase and lipase).

Lung injury is the secondary effect of pancreatitis most related to mortality in patients [32]. In the model of L-arginine-induced pancreatitis, lung MPO activity was increased 72 h after induction, corroborating previous findings [9]. The fact that the treatment with HEVm reduced lung MPO activity is complementary to the above-mentioned extrapancreatic protective effects, which might be a consequence of the reduction of pancreatic damage. Pancreatitis-related lung injury is multifactorial and is associated with many factors, such as the presence of activated enzymes, destruction of the lung surfactant, low lung perfusion rates, and increased cytokine in lung tissue [33]. Interestingly, in agreement with the decrease in lung MPO activity, we detected lower TNF-*α*, IL-1*β*, and IL-6 levels in lungs of animals treated with HEVm in comparison to the vehicle-treated group.

Since the HEVm is rich in flavanols and other polyphenols and these compounds have antioxidant effects [11, 34, 35], we speculated that the beneficial actions observed in pancreatic and extrapancreatic parameters could take place with the involvement of antioxidant activity. In fact, increased TBARS and carbonyl groups in parallel with decreased NP-SH and total antioxidant capacity (FRAP) in both pancreas and lung indicated that oxidative stress plays a significant role in this model, as previously shown [11, 36]. Treatment with HEVm maintained antioxidant capacity of the tissue (NP-SH and FRAP) and consequently reduced the oxidation markers (MDA and carbonyl), which can be attributed to the direct antioxidant effect of polyphenols found in HEVm [11, 22, 23, 34, 37]. Another reasonable possibility of explaining the antioxidant effect of HEVm is modulation of the activity of endogenous antioxidant enzymes. Pancreatitis induction was characterized by reduction of the activity of CAT, SOD, and GPx in pancreas and lung, corroborating previous findings [11]. The CAT and SOD activities were markedly altered by the treatment with HEVm, indicating that HEVm strongly modulates oxidative stress. Interestingly, CAT and SOD activities seemed to be more susceptible to reversion in pancreas than in lung, which agrees with the idea that the reduction of pancreatic damage was important to the modulation of extrapancreatic effects. The beneficial modulation of CAT and SOD activities in animals treated with HEVm was more evident than GPx activity, which was not altered by treatment with HEVm. Indeed, HEVm countered the L-arginine-induced increase in oxidation and decrease in antioxidative capacity.

The antioxidant effects observed in the treatment with HEVm can be caused by the compounds present in this extract. The phenolic compounds found in HEVm, like anthocyanins, rutin, catechin and epicatechins, and other phenolic compounds (e.g., quercetin, kaempferol, ellagic acid, and p-coumaric acid), are present in cranberries (for review, see [21, 22]) and certainly contributed to the reduction of oxidative stress, which in turn decreased the pancreatic lesions and resulted in less severe extrapancreatic alterations in mice.

An important concern about cranberry extracts is regarding their safety. Many studies have found no toxicity after treatment with cranberry extracts for 6–10 weeks in experimental animals [5, 6, 38] or in humans [39, 40], but a recent study found small differences in the spleen and kidney function of the progeny of mice treated with cranberry extract during pregnancy [41]. Thus, more specific studies are needed to assure the safety of cranberry extracts.

Altogether, our results indicate a protective role of HEVm in the model of AP induced by L-arginine, by reducing pancreatic damage, hyperalgesia, and remote alterations through the key participation of the antioxidant capacity of this extract. These findings provide insights into the possible use of this extract in the treatment of this disease.

## Figures and Tables

**Table 1:** Phenolic compounds identified in the hydroethanolic extract of fruits of *Vaccinium macrocarpon* (HEVm) by HPLC-DAD-ESI-MS/MS.

Peak	Proposed structure	RT (min)	Molecular ion	MS^2^/MS^3^ (*m/z*)	Concentration^*∗∗*^
1	Catechin^*∗*^	14,9	291	273; 165; 139; 123	581,82 ± 30,50
2	Epicatechin^*∗*^	18,5	291	273; 165; 139; 123	95,70 ± 4,01
3	Quercetin-3-O-rutinoside	24,5	611	465; 303	37,89 ± 6,55
4	Epicatechin gallate^a^	24,8	441	289; 169	139,22 ± 5,07
5	Cyanidin-3-O-galactoside^b^	13.4	449	287	2,45 ± 0,45
6	Cyanidin-3-O-glucoside^*∗*^	14.4	449	287	11,87 ± 0,75
7	Cyanidin-coumaroyl-hexoside	14.7	595	449/287	6,69 ± 0,54

RT, retention time. Peaks are numbered according to Figures 1(a) and 1(b). ^*∗*^Compounds' identity was confirmed by comparison with RT of standards and mass fragment profile and UV-Vis absorption spectra. ^*∗∗*^Expressed as mean ± SD of mg/100 g of extract. ^a^Quantified as equivalent of epicatechin. ^b^Quantified as equivalent of cyanidin-3-O-glucoside. Compounds 1–3 and 4–7 were analyzed in positive mode; compound 4 was analyzed in negative mode.

**Table 2:** The hydroethanolic extract of fruits of *Vaccinium macrocarpon* (HEVm) reduces serum biochemical parameters in mice submitted to acute pancreatitis by L-arginine.

Parameter	Saline + vehicle	Saline + L-arginine	HEVm (50 mg/kg) + L-arginine	HEVm (100 mg/kg) + L-arginine	HEVm (200 mg/kg) + L-arginine	Dexa (5 mg/kg) + L-arginine
Amylase (U/dL)	222 ± 21	968 ± 114^a^	873 ± 44	763 ± 73	346 ± 16^e^	458 ± 132^d^
Lipase (U/dL)	69 ± 13	475 ± 102^a^	313 ± 23	370 ± 52	196 ± 26^d^	209 ± 26^c^
ALT (UI/L)	4.2 ± 0.3	9.8 ± 0.3^a^	8.2 ± 1.2	6.3 ± 0.5^c^	5.6 ± 0.9^c^	7.2 ± 0.5
AST (UI/L)	6.4 ± 0.2	11.6 ± 1.5^b^	7.2 ± 0.7	6.1 ± 0.5^e^	7.1 ± 0.5^d^	8.5 ± 0.5

Groups of mice (*n* = 5-6) were submitted to injection of L-arginine or saline, treated with HEVm (50, 100, or 200 mg/kg), dexamethasone (Dexa; 5 mg/kg), or vehicle (saline) and serum was collected after 72 h of induction. ^a^*p* < 0.01 and ^b^*p* < 0.05 versus the respective saline + vehicle group or ^c^*p* < 0.05, ^b^*p* < 0.01, and ^e^*p* < 0.001 versus the respective AP + vehicle group.

## Data Availability

The data used to support the findings of this study can be accessed upon request to the corresponding author.

## Abbreviations

ALT: Alanine aminotransferase
AP: Acute pancreatitis
AST: Aspartate aminotransferase
CAT: Catalase
ELISA: Enzyme-linked immunosorbent assay
ESI: Electrospray ionization
FRAP: Ferric reducing/antioxidant power
GPx: Glutathione peroxidase
HEVm: Hydroethanolic extract of* Vaccinium macrocarpon*
HPLC-DAD: High-performance liquid chromatography coupled to the detector diode array
IL: Interleukin
MDA: Malondialdehyde
MPO: Myeloperoxidase
NP-SH: Nonprotein sulfhydryl groups
ROS: Reactive oxygen species
SOD: Superoxide dismutase
TNF-*α*: Tumor necrosis factor-*α*.
